# Interaction between low tidal volume ventilation strategy and severity of acute respiratory distress syndrome: a retrospective cohort study

**DOI:** 10.1186/s13054-019-2530-6

**Published:** 2019-07-12

**Authors:** Yanfei Shen, Guolong Cai, Shijin Gong, Lei Dong, Jing Yan, Wanru Cai

**Affiliations:** 1 Department of Intensive Care, Zhejiang Hospital, No. 12, Linyin Road, Hangzhou, 310000 Zhejiang People’s Republic of China; 2 Respiratory Department, The Second Affiliated Hospital of Zhejiang Chinese Medicine University, No. 318, Chaowang Road, Hangzhou, 310005 People’s Republic of China

**Keywords:** Acute respiratory distress syndrome, Low tidal volume, Mortality, PaO_2_/FiO_2_

## Abstract

**Background:**

Although low tidal volume is strongly recommended for acute respiratory distress syndrome (ARDS), whether or not the benefit varies according to the severity of ARDS remains unclear. This study aimed to investigate whether or not there is an interaction between low tidal volume and severity of ARDS.

**Methods:**

This was a secondary analysis from a randomized controlled trial. The patients were subgrouped according to whether the PaO_2_/FiO_2_ (P/F) was > 150 or ≤ 150 mmHg on day 0. The interaction between a tidal volume of 6 mL/kg and the P/F was investigated in hierarchical chi-square analysis and logistic regression models.

**Results:**

Eight hundred and thirty-six patients with ARDS were enrolled (345 in the high P/F subgroup [> 150 mmHg] and 491 in the low P/F subgroup [≤ 150 mmHg]). Compared to the traditional tidal volume group, the mortality of patients with low tidal volume was significantly lower in the high P/F subgroup (41/183 (22.4%) vs. 64/162 (39.5%), *p* = 0.001) but not in the low P/F subgroup (95/256 (37.1%) vs. 96/235 (40.8%), *p* = 0.414). In the hierarchical chi-square analysis, the test of homogeneity was significant (risk ratio of mortality 0.56 [0.40–0.79] vs. 0.91 [0.73–1.13], *p* = 0.018). In the multivariable logistic model, the odds ratio of mortality for the interacted item was significant (2.02, 95% confidence interval [CI] 1.06–3.86, *p* = 0.033). The odds ratio of mortality for low tidal volume was significant in the high P/F subgroup (0.42, 95% CI 0.24–0.72, *p* = 0.002) but not in the low P/F subgroup (0.89, 95% CI 0.60–1.31, *p* = 0.554).

**Conclusions:**

The benefits of low tidal volume ventilation remain uncertain in patients with severe ARDS. Further studies are needed to validate this significant interaction.

**Electronic supplementary material:**

The online version of this article (10.1186/s13054-019-2530-6) contains supplementary material, which is available to authorized users.

## Background

Acute respiratory distress syndrome (ARDS) is a devastating subtype of acute respiratory failure that presents as severe hypoxemia and bilateral pulmonary infiltrates. Globally, it affects more than 3 million patients annually and the overall mortality remains as high as 30–40% [1]. The cornerstone of management for ARDS is mechanical ventilation, with the aim of minimizing ventilator-induced lung injury (VILI). In 2000, a landmark trial from the ARDS Network [2] demonstrated that a low tidal volume using 6 mL/kg of predicted body weight (PBW) with a target plateau pressure below 30 cmH_2_O significantly reduced the mortality rate when compared with a traditional tidal volume strategy (12 mL/kg PBW). Subsequently, the results of other randomized controlled trials (RCTs) and meta-analyses [3–5] have demonstrated the efficacy of a low tidal volume strategy in reducing mortality in ARDS.

However, most of the previous studies [2, 6–9] have focused mainly on the efficacy of a reduced ventilation strategy in ARDS, and it has been unclear whether or not this benefit would be influenced by the severity of ARDS. Terragni et al. [10] found that patients with ARDS and lungs with larger collapsed areas were more prone to VILI despite a tidal volume limitation of 6 mL/kg PBW than those who had lungs with smaller areas of collapse, which was partly caused by an uneven distribution of tidal volume because the normal alveoli are more prone to distention than those that are collapsed. This raised the important but uninvestigated question of how much benefit patients with severe ARDS can derive from a tidal volume ventilation of 6 mL/kg PBW. Furthermore, we noticed in the RCTs reporting a negative effect of a low tidal volume ventilation strategy in ARDS [6, 7] that the baseline PaO_2_/FiO_2_ (P/F) value was significantly lower in the low tidal volume group (150 vs. 129 in the study by Brower et al. [6] and 145 vs. 123 in the study by Stewart et al. [7]). The reason underlying these inconsistent results remains unclear. However, they would be explicable if patients with severe ARDS do not actually benefit from ventilation with a tidal volume of 6 mL/kg. Accordingly, we performed a second analysis to investigate the interaction effect between ventilation with a low tidal volume and disease severity in patients with ARDS.

## Methods

### Data source

This was a secondary analysis of a prospective RCT [2] that was performed in 10 centers within the Acute Respiratory Distress Syndrome Network of the National Heart, Lung, and Blood Institute research network. The dataset included data for 902 patients, including 861 patients who were randomized to either 6 or 12 ml/kg in the tidal volume trial and an additional 41 patients who received 6 mL/kg in a study that compared lisofylline with placebo. The original study was approved by the institutional review board at each study center, and informed consent was obtained from the patients or their surrogates. All the data used in this study were approved by the Biologic Specimen and Data Repository Information Coordinating Center (BioLINCC, https://biolincc.nhlbi.nih.gov). The present study was approved by the institutional review board of Dongyang People’s Hospital. All the information was de-identified in the downloaded dataset.

### Inclusion and exclusion criteria

Patients under invasive mechanical ventilation support were screened if they met the following Berlin criteria: an acute decrease in the ratio of partial pressure of arterial oxygen to fraction of inspired oxygen (P/F) to ≤ 300, bilateral pulmonary infiltrates on a chest radiograph, and no clinical evidence of left atrial hypertension or a pulmonary capillary wedge pressure ≤ 18 mmHg. Patients were excluded if they were younger than 18 years of age, if they had participated in another trial in the 30 days before screening, if they were pregnant, or if they had other clinical conditions that could impair breathing or aggravate their clinical condition, such as neuromuscular disease, severe chronic respiratory disease, and high intracranial pressure (that may be worsened by hypercapnia). A detailed description of the inclusion and exclusion criteria is available in the original report [2].

### Data extraction

Demographic data, including age, body mass index, sex, and ethnicity, were collected, as well as information on comorbidities, such as diabetes, immunosuppression, and leukemia. Biochemical measurements, including white blood cell count, platelet count, serum creatinine, albumin, sodium, and bilirubin, and the plasma glucose level were also extracted. Other variables, such as fluid balance, radiographic acute lung injury (ALI) score, pneumothorax, and the P/F, were recorded. Given that the aim of the study was to investigate the interaction between P/F and low tidal volume ventilation in ARDS, two versions of the P/F value at different time points were used for robustness, i.e., the P/F at the time of screening, which was recorded at the time of screening in ICUs with mechanical ventilation and settings selected by clinicians, and on day 0 of the original trial which was recorded on the first day of the original trial with protocolized ventilator settings. The sensitivity analysis was performed using the P/F value at the time of screening. For accuracy, the missing P/F value was not inputted and patients without a P/F on day 0 were excluded.

### Definition of ventilation procedures

The original study compared the effect of low and traditional tidal volume in ARDS. In the traditional group, the tidal volume was set at 12 mL/kg PBW with a target plateau pressure (airway pressure measured after a 0.5-s pause at the end of inspiration) set at ≤ 50 cmH_2_O by stepwise reduction of tidal volume (1 mL/kg PBW per decrement) if necessary. In the low tidal volume group, the tidal volume was set at 6 mL/kg PBW with the plateau pressure at ≤ 30 cmH_2_O by stepwise reduction of the tidal volume (1 mL/kg PBW per decrement) if necessary. The minimal tidal volume was 4 mL/kg PBW. The volume assist control mode was used in the two groups until weaning or 28 days after randomization, whichever came first. All other ventilation procedures, including weaning, were identical in the two groups. A more detailed description is available in the original report [2].

### Study endpoint

In the original study, the endpoints were divided into three categories: (1) the proportion of patients who went home with unassisted breathing, (2) the proportion of patients who died before discharge to home with unassisted breathing or died before achieving unassisted breathing for 48 h, and (3) the proportion of patients meeting neither of these two conditions. The patient status was checked at intervals of ≤ 30 days until condition 1 or 2 occurred, with a maximum duration of 180 days. Patients who met condition 2 were reported as non-survivors, and those who met the other conditions were reported as survivors.

### Statistical analysis

Continuous variables were expressed as the mean ± standard deviation or median (interquartile range) as appropriate. The Student’s *t* test and Wilcoxon rank-sum test were used as appropriate. Categorical data were expressed as proportions and compared using the chi-square test. The included patients were divided into two subgroups according to whether or the P/F value on day 0 was > 150 mmHg or ≤ 150 mmHg. Hierarchical chi-square analysis was used to test for homogeneity between the two subgroups. Multivariable logistic regression was used for covariate adjustment. The logistic models were built using the stepwise backward method as follows. First, variables identified to have a *p* value less than 0.20 in the univariate analysis were included for further multivariable analysis. Nine covariables were identified in this step: age, maximum respiratory rate, immunosuppression, leukemia, radiographic ALI score, lowest platelet count, highest creatinine level, and fluid balance. Next, we used a stepwise backward elimination method to remove variables with a *p* value > 0.1 (serum creatinine was removed in this step). Multicollinearity was tested using the variance inflation factor (VIF) method (the radiographic ALI score had a VIF ≥ 5 so was removed). Goodness of fit tests were applied to all logistic regression models. The interaction effect between the P/F and low tidal volume was tested by adding an interacted item (P/F * low tidal volume) in the above model. Predictive marginal effects of low tidal volume were estimated for interpretation at different P/F values on day 0. A two-tailed test was performed, and a *p* value < 0.05 was considered statistically significant. All statistical analyses were performed using Stata 11.2 (StataCorp, College Station, TX, USA).

## Results

Data for 902 patients were available in the dataset downloaded from BioLINCC. The P/F value on day 0 was missing in 66 patients, so these patients were excluded from the analysis. Finally, 836 patients (540 survivors and 296 non-survivors) were included in the study. These patients were divided into two subgroups according to whether or not their P/F on day 0 was > 150 mmHg (the high P/F subgroup, *n* = 345) or ≤ 150 mmHg (the low P/F subgroup, *n* = 491). No significant differences were detected in the demographics or in the physical or biochemical parameters at baseline, so the characteristics of the group that underwent a low tidal volume procedure and those of the group that underwent a traditional tidal volume procedure were considered well balanced (Table 1).

**Table 1:** Comparison of baseline characteristics between the traditional and low tidal volume subgroups

Variables	Subgroup with PaO_2_/FiO_2_ > 150			Subgroup with PaO_2_/FiO_2_ ≤ 150		
	Traditional tidal volume (*n* = 162)	Low tidal volume (*n* = 183)	*p* value	Traditional tidal volume (*n* = 235)	Low tidal volume (*n* = 256)	*p* value
Age (years)	50.3 ± 18.5	50.8 ± 16.8	0.800	52.5 ± 17.3	50.7 ± 16.6	0.233
Male [*n* (%)]	95 (58.6)	117 (63.9)	0.317	138 (58.7)	148 (57.8)	0.838
BMI (kg/m^2^)	27.2 ± 7.0	27.4 ± 7.6	0.803	26.8 ± 6.4	27.4 ± 6.59	0.273
Ethnicity (White, %)	119 (73.4)	138 (75.4)	0.678	162 (68.9)	190 (74.2)	0.194
Ethnicity (Black, %)	31 (19.1)	28 (15.3)	0.345	42 (17.8)	41 (16.0)	0.583
Comorbidities						
Chronic dialysis [*n* (%)]	6 (3.7)	3 (1.6)	0.226	4 (1.7)	6 (2.3)	0.620
Leukemia [*n* (%)]	4 (2.4)	3 (1.6)	0.579	5 (2.1)	7 (2.7)	0.669
Immunosuppression [*n* (%)]	17 (10.4)	13 (7.1)	0.256	24 (10.2)	32 (12.6)	0.436
Diabetes [*n* (%)]	22 (13.5)	25 (13.6)	0.997	33 (14.0)	36 (14.0)	0.973
Elective surgery [*n* (%)]	12 (7.4)	17 (9.2)	0.520	18 (7.6)	26 (10.1)	0.371
Radiographic ALI score median (IQR)	4 (3–4)	4 (3–4)	0.671	4 (4–4)	4 (4–4)	0.650
Pneumothoraces [*n* (%)]	12 (7.4)	16 (8.7)	0.687	44 (18.7)	35 (13.6)	0.117
Chest tube [*n* (%)]	42 (25.9)	38 (20.7)	0.218	57 (24.2)	71 (27.7)	0.412
Parameters on screen						
PaO_2_/FiO_2_ (mmHg)	161.5 ± 59.2	165.0 ± 65.8	0.601	103.8 ± 39.1	107.2 ± 41.6	0.358
PaO_2_ (mmHg)	103.9 ± 49.2	101.7 ± 43.5	0.677	81.4 ± 32.4	79.1 ± 27.8	0.400
Indexes within 24-h preceding trial						
Maximum respiratory rate	31.3 ± 12.4	28.9 ± 10.0	0.053	30.5 ± 10.7	30.8 ± 10.7	0.748
Minimum mean blood pressure (mmHg)	63.5 ± 14.3	63.7 ± 14.2	0.862	61.1 ± 13.5	59.8 ± 11.1	0.220
Maximum serum creatinine (mg/dL)	1.76 ± 1.68	1.76 ± 1.47	0.979	1.75 ± 1.61	1.54 ± 1.37	0.129
Minimum platelet count (10^9^/L)	154.3 ± 108.9	145.7 ± 98.7	0.439	157.9 ± 108.9	176.6 ± 135.2	0.094
Maximum white blood cell (10^9^/L)	15.6 ± 10.6	14.5 ± 8.8	0.272	14.6 ± 8.9	15.2 ± 11.8	0.587
Parameters on day 0 of the trial						
Fluid intake (mL)	5217.7 ± 3782.1	5065.2 ± 4070.9	0.719	4896.0 ± 4045.2	5366.1 ± 4293.9	0.213
Fluid output (mL)	2374.0 ± 1716.2	2473.2 ± 1698.6	0.590	2375.6 ± 1694.9	2396.8 ± 1630.3	0.887
PaO_2_/FiO_2_ (mmHg)	212.2 ± 65.2	210.6 ± 56.0	0.817	103.2 ± 26.7	104.2 ± 26.5	0.684
PEEP on day 0 (cmH_2_O)	7.4 ± 3.8	7.4 ± 3.2	0.999	8.9 ± 3.9	9.5 ± 3.9	0.109
PEEP on day 1 (cmH_2_O)	6.5 ± 3.2 (*n* = 162)	6.8 ± 2.3 (*n* = 179)	0.294	9.6 ± 3.3 (*n* = 233)	11.1 ± 3.7 (*n* = 251)	< 0.001
Plateau pressures on day 1 (cmH_2_O)	28.8 ± 7.6 (*n* = 125)	27.3 ± 7.3 (*n* = 137)	0.086	31.6 ± 8.2 (*n* = 186)	31.6 ± 7.6 (*n* = 211)	0.975
Plateau pressures on day 2 (cmH_2_O)	30.6 ± 8.4 (*n* = 146)	22.5 ± 5.8 (*n* = 151)	< 0.001	33.7 ± 8.6 (*n* = 223)	26.4 ± 6.4 (*n* = 239)	< 0.001
Driving pressure on day 1 (cmH_2_O)	21.6 ± 6.9 (*n* = 125)	19.7 ± 6.9 (*n* = 137)	0.027	22.6 ± 7.2 (n = 186)	21.8 ± 6.7 (n = 211)	0.276
Driving pressure on day 2 (cmH_2_O)	23.9 ± 7.2 (*n* = 146)	15.4 ± 5.3 (*n* = 151)	< 0.001	23.9 ± 7.3 (*n* = 223)	15.3 ± 5.3 (*n* = 239)	< 0.001

*Abbreviations*: *BMI* body mass index, *IQR* interquartile range, *PEEP* positive end expiratory pressure

The baseline P/F value on day 0 was also similar between the two tidal volume groups (212.2 ± 65.2 vs. 210.6 ± 56.0 in the high P/F subgroup, *p* = 0.817; 103.2 ± 26.7 vs. 104.2 ± 26.5 in the low P/F subgroup, *p* = 0.684). Therefore, hierarchical chi-square analysis was used to evaluate the effect of low tidal volume in these two subgroups. Compared to the traditional tidal volume group, the mortality of patients with low tidal volume was significantly lower in the high P/F subgroup (41/183 (22.4%) vs. 64/162 (39.5%), *p* = 0.001) but not in the low P/F subgroup (95/256 (37.1%) vs. 96/235 (40.8%), *p* = 0.414). In the hierarchical chi-square analysis, the test for homogeneity for the risk ratio of mortality in these two subgroups was significant (0.56 [0.40–0.79] vs. 0.91 [0.73–1.13], *p* = 0.018) (Table 2).

**Table 2:** Crude outcomes and test of homogeneity in the traditional and low tidal volume subgroups

Variables	Subgroup with PaO_2_/FiO_2_ > 150			Subgroup with PaO_2/_FiO_2_ ≤ 150		
	Traditional tidal volume (*n* = 162)	Low tidal volume (*n* = 183)	*p*	Traditional tidal volume (*n* = 235)	Low tidal volume (*n* = 256)	*p*
Patients achieving unassisted breathing for 48 h [*n* (%)]	96 (59.2)	141 (77.0)	< 0.001	119 (50.6)	151 (58.9)	0.063
Death before discharge home and breathing without assistance [*n* (%)]	64 (39.5)	41 (22.4)	0.001	96 (40.8)	95 (37.1)	0.414
Risk ratio of death^#^	0.56 (0.40–0.79)			0.91 (0.73–1.13)		

^#^The risk ratio was calculated using hierarchical chi-square analysis, and the *p* value for homogeneity (Mantel-Haenszel) was 0.0185

**Fig. 1:**
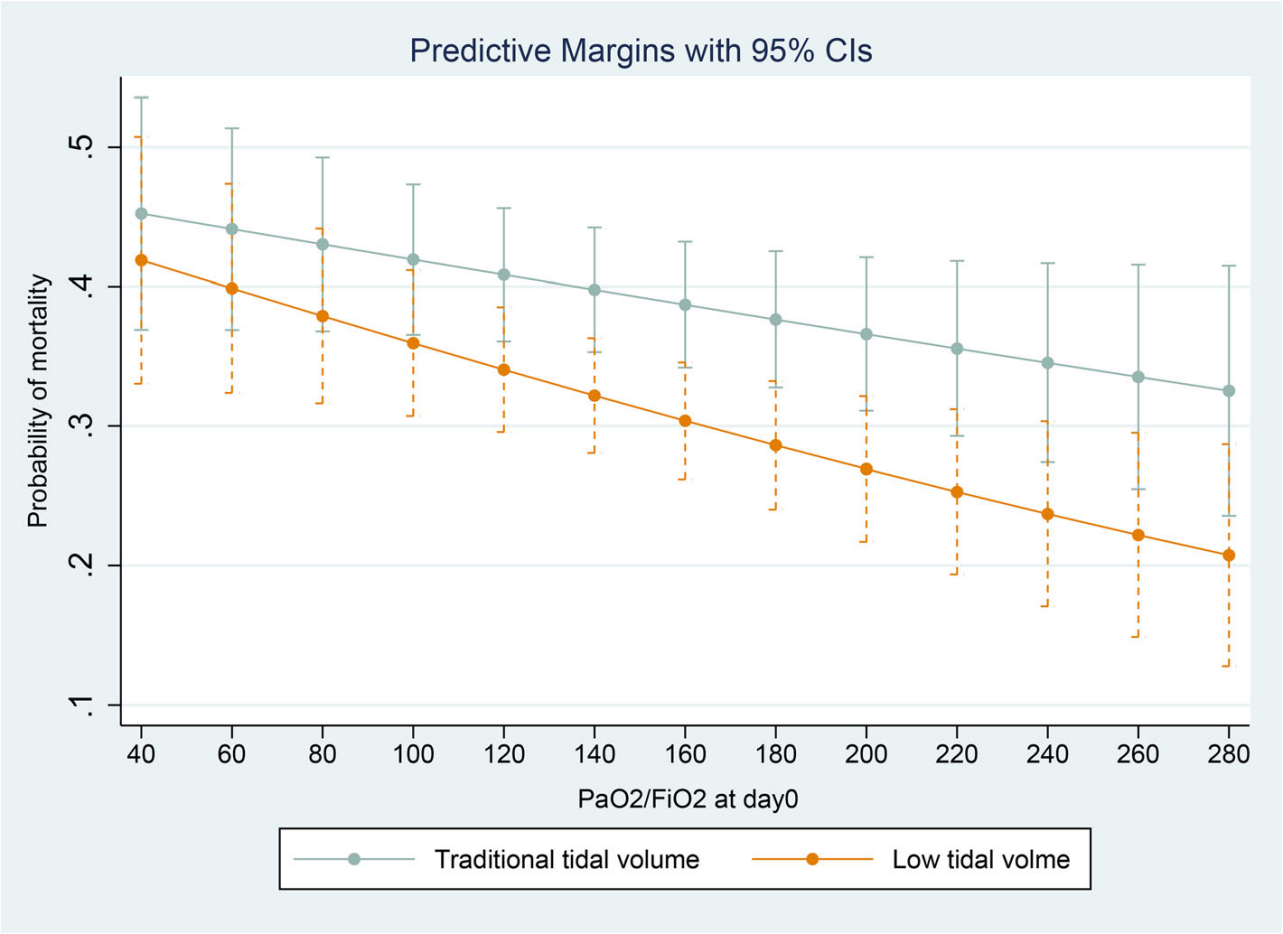
Predictive marginal effect of low tidal volume strategy at different PaO_2_/FiO_2_ at day 0. PaO_2_/FiO_2_, ratio of partial pressure of arterial oxygen to fraction of inspired oxygen

Given the retrospective nature of the research, there remained a risk that this finding was biased by unadjusted covariables. Therefore, multivariable logistic regression was applied for adjustment, and the interactive effect was evaluated by adding the interacted item (P/F * low tidal volume) in the model. The odds ratio of mortality for the interacted item was significant (2.02, 95% confidence interval [CI] 1.06–3.86, *p* = 0.033; in Additional file 1: Table S1), suggesting a P/F-dependent effect of low tidal volume. In the subgroup analysis (Table 3), the odds ratio of mortality for low tidal volume was significant in the high P/F subgroup (0.42, 95% CI 0.24–0.72, *p* = 0.002) but not in the low P/F subgroup (0.89, 95% CI 0.60–1.31, *p* = 0.554). The predicted marginal effect on mortality of low tidal volume was also estimated at different P/F values on day 0 (40, 60, 80, 100, 120, 140, 160, 180, 200, 220, 240, 260, 280) in Fig. 1. The difference in the probability of mortality between the traditional and low tidal volume groups increased with increasing P/F on day 0, which was consistent with the previous findings.

**Table 3:** Multivariable logistic regression in the traditional and low tidal volume subgroups

Variables	Model 1 Subgroup with PaO_2_/FiO_2_ > 150 (*n* = 345)		Model 2 Subgroup with PaO_2_/FiO_2_ ≤ 150 (*n* = 491)	
	Adjusted odds ratio (95% CI)	*p*	Adjusted odds ratio (95% CI)	*p*
Low tidal volume	0.42 (0.24–0.72)	0.002	0.88 (0.60–1.31)	0.554
Immunosuppression	3.60 (1.38–9.40)	0.009	1.42 (0.75–2.67)	0.274
Leukemia	0.87 (0.14–5.19)	0.887	5.52 (1.23–24.8)	0.026
Respiratory rate	1.02 (1.00–1.05)	0.032	1.02 (1.00–1.04)	0.008
Platelet count (10^9^/L)	0.99 (0.99–0.99)	0.011	0.99 (0.99–1.00)	0.106
Age (years)	1.05 (1.03–1.06)	< 0.001	1.04 (1.02–1.05)	< 0.001
Fluid balance	1.10 (1.04–1.16)	0.001	1.05 (1.00–1.10)	< 0.001

Note: The *p* value for interaction item (PaO2/FiO2 * low tidal volume) was 0.033 in Additional file 1: Table S1. The VIF value were 2.91 and 2.82, and the *p* values of goodness of fit are 0.479 and 0.355 for model 1 and model 2, respectively

Eight hundred and thirty-one patients were included in the sensitivity analysis using the P/F value at screening, and the results were similar to the main finding that the effect on mortality of low tidal volume was significant in patients with a P/F > 150 mmHg (43/110 (39.1%) vs. 32/145 (22.1%), *p* = 0.003) but not in patients with a P/F ≤ 150 mmHg (116/285 (40.7%) vs. 104/291 (35.7), *p* = 0.220). However, only 255 patients were included in the high P/F subgroup, and the *p* value for homogeneity was less significant (0.047 in the hierarchical chi-square analysis and 0.064 in the logistic model, in Additional file 1: Tables S2 and S3, respectively).

## Discussion

The main finding of our study is the significant interaction between low tidal volume ventilation and oxygenation in patients with ARDS. In patients with a P/F > 150 mmHg, a ventilation strategy that included a tidal volume of 6 mL/kg PBW with a target plateau pressure at 30 cmH_2_O resulted in an absolute 17.1% reduction in mortality; however, the benefit may have been weakened in patients with a P/F value ≤ 150 mmHg. Despite the efficacy of the low tidal volume strategy having already been investigated in many studies, our finding proposes a new but important concept that needs to be investigated further.

Whether or not use of lower tidal volume ventilation can reduce the risk of VILI has been investigated for the past 30 years, which is recommended in the current ARDS consensus [11, 12]. However, the extent to which tidal volume and inspiratory airway pressure should be reduced to optimize clinical outcomes remains controversial. Most of the previous trials focused on the efficacy of a reduced tidal volume strategy in patients with ARDS, and whether or not this benefit would be influenced by the severity of ARDS has remained unclear. However, the previous research demonstrated a heterogeneous distribution of pulmonary alterations in ARDS on computed tomography images, such as hyperinflated, normally aerated, and nonaerated compartments [13, 14], which were associated with different clinical outcomes [15]. Pathophysiologically, this heterogeneity may lead to an uneven distribution of tidal volume, given that the nonaerated compartments would be stiffer than the other compartments and have decreased compliance. Consequently, alveoli in the relatively normally aerated area are prone to more hyperinflation than other areas, which may play a role in the mechanism of VILI. Involvement of alveoli in normally aerated areas in the mechanism is consistent with the finding by Terragni et al. [10] that patients with ARDS with more nonaerated compartments were more likely to develop VILI than those with fewer nonaerated compartments. Furthermore, this phenomenon is also partly explained by our finding that the benefit of 6 mL/kg PBW ventilation may be reduced in patients with low P/F because a low P/F to a certain extent represents more severe lung status and may be associated with more nonaerated compartments in which VILI may be more likely to occur. On the other hand, Terragni et al. [16] also found that the risk of tidal hyperinflation and pulmonary inflammation was lower with an ultra-low tidal volume strategy (4 ml/kg PBW) than with the 6 mL/kg PBW ventilation strategy but could be accompanied by development of severe respiratory acidosis necessitating extracorporeal carbon dioxide removal [2]. Given that removal of extracorporeal carbon dioxide in all patients with ARDS would be clinically impracticable, identifying patients who would or would not benefit from ventilation with a tidal volume of 6 mL/kg PBW became critically important. In the present study, we found that a low tidal volume of 6 mL/kg PBW had a heterogeneous effect according to the severity of ARDS. Unlike in previous studies, we found that the effect of a low tidal volume of 6 mL/kg PBW was uncertain in patients with severe ARDS (P/F ≤ 150) but was significant in those with mild ARDS. It is possible that the mechanism underlying this interaction is multifactorial. For instance, a larger nonaerated compartment may to some degree represent more severe ARDS that would be associated with a lower P/F. Therefore, in theory, compared with patients with high P/F, those with low P/F may benefit less from a low tidal ventilation strategy because these are the patients who are more likely to develop VILI. If this is the case, the conclusions of previous three RCTs [6–8] that found no significant benefit of low tidal volume in patients with ARDS would be understandable. One common issue in those studies was that the baseline P/F value was lower in the low tidal ventilation groups (150 vs. 129 in the study by Brower et al. [6], 145 vs. 123 in the study by Stewart et al. [7], and 144 vs. 155 in the study by Brochard et al. [8]). Therefore, the effect of low tidal volume may be weakened in low tidal volume groups, leading to these nonsignificant conclusions.

Several limitations of this study need to be addressed. First, despite the large sample size, the statistical power of the study was significantly weakened by the subgroup analysis. Therefore, the nonsignificant finding in the low P/F subgroup needs further investigation. Second, during the last decade, other mechanical parameters, such as transpulmonary pressure and lung compliance, have been reported to be closely associated with clinical outcomes in patients with ARDS. However, these data are lacking in the current study, which increased the risk of bias. For example, transpulmonary pressure and lung compliance may vary more in patients with severe ARDS than in those with mild ARDS, which may offset the benefit of low tidal volume in the severe subgroup. Given the lack of data, the impact of these mechanical properties cannot be inferred from the present study. Third, severe ARDS was defined as P/F ≤ 150 mmHg in the current study, and this cut-off value was also adopted in previous studies [17, 18]. The reason we did not use the Berlin definition (100 mmHg as the cut-off value) is that the proportion of patients with mild ARDS was very small and not large enough for statistical analysis.

## Conclusions

Our present findings suggest that the effects of a lower tidal volume ventilation strategy (6 mL/kg with a plateau pressure goal of 30 cmH2O) may be smaller in patients with more severe ARDS than in those with a P/F > 150 mmHg. Further studies that account for all the other factors that might potentially influence the outcome are needed before firm conclusions to be drawn regarding the effects of low tidal volumes.

## Additional file

Additional file 1: **Table S1.** Interaction between low tidal volume and PaO_2_/FiO_2_ in multivariable logistic regression. **Table S2.** Crude outcomes test of homogeneity in the subgroups with high and low PaO_2_/FiO_2_. (DOC 58 kb)

## Data Availability

The datasets presented in the current study are available in the BioLINCC website. (https://biolincc.nhlbi.nih.gov).

## Abbreviations

ALI: Acute lung injury
ARDS: Acute respiratory distress syndrome
CI: Confidence interval
PaO2/FiO2: Partial pressure of arterial oxygen to fraction of inspired oxygen
PBW: Predicted body weight
RCT: Randomized controlled trial
VIF: Variance inflation factor
VILI: Ventilator-induced lung injury
