# Supplementary material for: Interaction between low tidal volume ventilation strategy and severity of acute respiratory distress syndrome: a retrospective cohort study
Source: Crit Care. 2019 Jul 12;23:254. doi: 10.1186/s13054-019-2530-6 (PMC6626332; doi:10.1186/s13054-019-2530-6)
Supplement: Supplementary file 1 — Table S1. Interaction between low tidal volume and PaO2/FiO2 in multivariable logistic regression. Table S2. Crude outcomes test of homogeneity in the subgroups with high and low PaO2/FiO2. (DOC 58 kb) [file 13054_2019_2530_MOESM1_ESM.doc]

**Additional file 1**

Table S1 Interaction between low tidal volume and PaO_2_/FiO_2_ in multivariable logistic regression

|  | **Model 1 (n = 836)** | | **Model 2 (n = 836)** | |
| --- | --- | --- | --- | --- |
| **Variable** | Adjusted OR (95% CI) | p-value | Adjusted OR (95% CI) | p-value |
| **Low tidal volume** | 0.67 (0.49–0.92) | 0.013 | 0.88 (0.59–1.31) | 0.542 |
| **PaO_2_/FiO_2_ at day 0 >150** | 0.66 (0.48–0.91) | 0.013 | 0.94 (0.60–1.47) | 0.790 |
| **Immunosuppression** | 1.95 (1.16–3.27) | 0.012 | 1.90 (1.13–3.21) | 0.015 |
| **Leukemia** | 2.80 (0.91–8.68) | 0.072 | 2.80 (0.90–8.71) | 0.074 |
| **Respiratory rate** | 1.02 (1.00–1.04) | < 0.001 | 1.02 (1.01–1.03) | 0.001 |
| **Platelet count (10×9/L)** | 0.99 (0.99–0.99) | 0.007 | 0.99 (0.99–0.99) | 0.005 |
| **Age (years)** | 1.04 (1.03–1.06) | < 0.001 | 1.04 (1.03–1.05) | < 0.001 |
| **Fluid balance** | 1.07 (1.03–1.11) | 0.001 | 1.07 (1.03–1.11) | 0.001 |
| **(Low tidal volume) * (PaO_2_/FiO_2_ >150)** | N/A |  | 0.49 (0.25–0.94) | 0.033 |

Notes: The interacted item [(low tidal volume) * (PaO_2_/FiO_2_ >150)) was only included in model 2 and the p-value was significant. The VIF values are 2.72 and 3.13 and the p-values for goodness of fit are 0.407 and 0.449 for models 1 and 2, respectively. CI, confidence interval; OR, odds ratio

Table S2 Crude outcomes test of homogeneity in the subgroups with high and low PaO_2_/FiO_2_

|  | **Subgroup with PaO_2_/FiO_2_ (screen) >150** | | | **Subgroup with PaO_2_/FiO_2_ ≤150** | | |
| --- | --- | --- | --- | --- | --- | --- |
| **Variable** | High tidal volume  (n=110) | Low tidal volume  (n=145) | p-value | High tidal volume  (n=285) | Low tidal volume  (n=291) | p-value |
| **Death before discharge home and breathing without assistance [n (%)]** | 43 (39.0) | 32 (22.0) | 0.003 | 116 (40.7) | 104 (35.7) | 0.220 |
| **Risk ratio of death^#^** | 0.56 (0.38–0.82) | |  | 0.88 (0.71–1.08) | |  |

Notes: A total of 831 patients were included in this sensitivity analysis. The patients were divided into two subgroups according to the PaO_2_/FiO_2_ at screening.

#The risk ratio was calculated using hierarchical chi-square analysis and the p-value for homogeneity (Mantel-Haenszel) was 0.047.

Table S3 Multivariable logistic regression in the subgroups with high and low PaO_2_/FiO_2_

|  | **Model 1**  **Subgroup with PaO_2_/FiO_2_ >150 (n=255)** | | **Model 2**  **Subgroup with PaO_2_/FiO_2_ ≤150 (n=576)** | |
| --- | --- | --- | --- | --- |
| **Variables** | Adjusted OR (95% CI) | p-value | Adjusted OR (95% CI) | p-value |
| **Low tidal volume** | 0.46 (0.25–0.85) | 0.013 | 0.79 (0.55 – 1.15) | 0.230 |
| **Immunosuppression** | 7.26 (2.20–23.98) | 0.001 | 1.30 (0.72 – 2.37) | 0.375 |
| **Leukemia** | 0.89 (0.09–8.86) | 0.926 | 3.82 (1.03 – 14.18) | 0.045 |
| **Respiratory rate** | 1.01 (0.98–1.04) | 0.230 | 1.02 (1.00 - 1.04) | 0.003 |
| **Platelet count (10×9/L)** | 0.99 (0.99–1.00) | 0.336 | 0.99 (0.99 – 0.99) | 0.011 |
| **Age (years)** | 1.04 (1.0 –1.06) | < 0.001 | 1.04 (1.02 – 1.05) | < 0.001 |
| **Fluid balance** | 1.06 (0.99–1.14) | 0.079 | 1.09 (1.03 – 1.14) | 0.001 |

Notes: The p-value for the interaction item (PaO_2_/FiO_2_ * low tidal volume) was 0.064 in Table S1. The VIF values were 2.81 and 2.86 and the p-values for goodness of fit are 0.540 and 0.339 for models 1 and 2, respectively. CI, confidence interval; OR, odds ratio
